# Current use of autologous adipose tissue-derived stromal vascular fraction cells for orthopedic applications

**DOI:** 10.1186/s12929-017-0318-z

**Published:** 2017-01-31

**Authors:** Jaewoo Pak, Jung Hun Lee, Kwang Seung Park, Moonhee Park, Lin-Woo Kang, Sang Hee Lee

**Affiliations:** 1 Stems Medical Clinic, 32-3 Chungdamdong, Gangnamgu, Seoul, 06068 Republic of Korea; 2 TEDA-Puhua International Hospital, Tianjin, 300457 People’s Republic of China; 3 Life Science Institute, Komplek Permata Senayan, Jalan Tentara Pelajar, Jakarta Selatan, 12210 Indonesia; 4 National Leading Research Laboratory, Department of Biological Sciences, Myongji University, 116 Myongjiro, Yongin, Gyeonggido 17058 Republic of Korea; 5 DNA Analysis Division, Seoul institute, National Forensic Service, 139 Jiyangro, Yangcheongu, Seoul, 08036 Republic of Korea; 6 Department of Biological Sciences, Konkuk University, 1 Hwayangdong, Gwangjingu, Seoul, 05029 Republic of Korea

**Keywords:** Mesenchymal stem cell, Stromal vascular fraction, Autologous adipose tissue-derived stem cells, Effectiveness and safety, Orthopedic applications

## Abstract

Autologous adipose stromal vascular fractions (SVFs) containing adipose tissue-derived stem cells (ASCs) are currently being used in clinical settings for various orthopedic applications for human patients. Due to its potential capability of regenerating cartilage, bone, and tendons, autologous adipose SVFs are being tried in treating patients with osteoarthritis (OA), chondromalacia, meniscus tear, osteonecrosis of the femoral head, and tendon injuries. Here, we have reviewed available human clinical studies with regard to patient applications of autologous adipose SVF containing ASCs, specifically assessing effectiveness and safety in the field of orthopedic disorders. All studies reviewed in this article presents potential benefits of autologous adipose SVF in various orthopedic applications without any serious side effects.

## Background

Musculoskeletal injuries and damage are common health problems in both young and old patients [1]. Various treatment modalities are available for such musculoskeletal injuries. However, most of these modalities provide only symptomatic relief [2]. The regenerative potential of injured and damaged tissue with stem cells is a promising new treatment strategy in the field of orthopedics. Stem cells can be categorized into two major forms: embryonic stem cells and adult stem cells [3]. Adult stem cells, which include mesenchymal stem cells (MSCs), can be further divided into non-culture expanded forms, also known as stromal vascular fractions (SVF), and culture expanded forms [3]. Often, the SVFs are autologous in nature and the process of obtaining SVFs may require a procedure with a physician. On the contrary, culture expanded stem cells involve cell growth and cell expansion using various nutrients in a laboratory setting. Thus, culture expanded stem cells are usually considered to be a pharmaceutical product requiring government regulatory clearance and approval in Korea [4]. Due to such government regulatory issues, adipose SVF has been more commonly used for various orthopedic applications in clinical settings. Currently two common forms of SVFs are readily available: bone marrow and adipose tissue [5].

Although MSCs can be found in numerous human tissues, a clinically applicable quantity of autologous non-culture expanded MSCs can be obtained only from bone marrow and adipose tissue [5, 6]. MSCs contained in adipose tissue are called adipose tissue-derived stem cells (ASCs) and are considered to be one specific type of MSCs, and they have been shown to differentiate into bones and cartilage [5–9]. In 2001 and 2002, Zuk et al. showed that adipose tissue contains MSCs in SVF and that these MSCs have the capacity to differentiate into cartilage and bone [8, 9]. The earliest clinical application of autologous adipose SVF with one surgical procedure to treat widespread traumatic calvarial defects was reported in 2004 by Lendeckel et al. [10].

In 2011, Pak had successfully used autologous adipose SVF for cartilage and bone regeneration in human patients without a surgical procedure [11]. Afterward, numerous clinical studies have been published about OA treatment with autologous adipose SVF. We conducted a literature search in the PubMed, Medline, and Embase. We used the keywords as our search terms. We combined terms for selected indications (stromal vascular fraction, stem cell, orthopedic, and adipose). The literature search included all studies published in English between 2010 and 2016. The criteria for the inclusion of studies in our review encompassed clinical studies on autologous adipose SVF injection conducted on humans for orthopedic applications. These studies will be reviewed in this article and summarized in Table 1.

**Table 1:** Current use of adipose SVF containing ASCs for orthopedic applications

Study (yr)	Intervention treatment	Study type	Number of subjects and diseases	Subject characteristic [age (yr); gender]	Previous therapy	Concurrent treatment	Follow-up (mo)	Outcome measures	Results	Authors’ conclusion
Pak (2011) [11]	Adipose SVF (ASC) + PRP + HA via percutaneous injections	Case report	2 OA	70 and 79; 2 F with chronic knee pain	Various conservative treatments without any success	None	3	VAS; functions (FRI, ROM); MRI	VAS/function improvements and MRI evidence of cartilage regeneration	ASC + PRP + HA: potentially effective in regenerating cartilage in humans
Pak et al. (2013) [18]	Adipose SVF (ASC) + PRP + HA via percutaneous injections	Retrospective cohort study	91 various orthopedic applications including OA	Mean 51.23 ± 1.50 (range, 18–78); 45 M and 46 F	Various conservative treatments without any success	None	26.62 ± 0.32	VAS; functions	Statistically significant improvement in both VAS and functions	ASC + PRP + HA: safe and potentially effective
Pak et al. (2016) [19]	Adipose SVF (ASC) + PRP + HA + ECM via percutaneous injections	Case report	3 OA	68; 1 M. 60 and 87; 2 F.	Various conservative treatments without any success	None	3.5	VAS; functions (FRI, ROM); MRI	VAS/function improvements and MRI evidence of cartilage regeneration	ASC + PRP + HA + ECM: potentially effective in regenerating cartilage in humans
Koh and Choi (2012) [20]	Adipose SVF (ASC) + PRP via percutaneous injections	Non-randomized, retrospective, comparative study: ASC + PRP vs PRP alone	25 OA Study group (ASC + PRP): 25; control group (PRP alone): 25	Study group: mean 54.1 (range, 34–69); 8 M and 17 F	Various treatments without any success	None	16.4	VAS; functions (Lysholm, Tegner)	ASC + PRP: more effective than PRP-control group	ASC + PRP: potentially effective in patients with cartilage defects
Koh et al. (2013) [21]	Adipose SVF (ASC) + PRP via percutaneous injections	Case series	18 OA	Mean 54.6; 6 M and 12 F	Various treatments without any success	Arthroscopic lavage before knee-fat-pad-derived adipose SVF + PRP injection	24.3	VAS; functions (WOMAC, Lysholm); MRI	VAS/function/MRI improvements	ASC + PRP: effective in treating OA of knees
Koh et al. (2014) [22]	Adipose SVF (ASC) + PRP under arthroscopic guidance	Case series	35 with 37 knee joints of OA	Mean 57.4 (range, 48–69); 14 M and 21 F	Various treatments without any success	Arthroscopic lavage before adipose SVF + PRP injection	12.7	VAS; functions; arthroscopy	94% patients had excellent clinical improvement; 76% had abnormal repair tissue.	Scaffolds may be needed to treat patients with large cartilage lesions.
Koh et al. (2014) [23]	Adipose SVF (ASC) + PRP under arthroscopic guidance	Comparative study: adipose SVF + PRP vs PRP only	44 OA	ND	Various treatments without any success	Open-wedge high tibial osteotomy	24	VAS; functions; arthroscopy	Adipose SVF + PRP is more effective than PRP alone.	ASC therapy, in conjunction with HTO, mildly improved cartilage healing and showed good clinical results compared with PRP only.
Koh et al. (2015) [24]	Adipose SVF (ASC) + PRP under arthroscopic guidance	Case series	30 for adipose SVF + PRP injection; 16 for second look arthroscopy for OA	Mean 70.3 (range, 65–80); 5 M and 25 F	Various treatments without any success	Arthroscopic lavage before ASC + PRP injection	24	VAS; functions; 2nd look arthroscopy	VAS/function improvements; improved and maintained cartilage status	ASC + PRP: effective in treating elderly patients with OA
Kim et al. (2015) [25]	Adipose SVF (ASC) under arthroscopic guidance	Comparative study: adipose SVF vs adipose SVF + fibrin glue (as a scaffold)	54 OA	Mean 57.5 ± 5.8; 22 M and 32 F	Various treatments without any success	None	28.6	VAS; functions; arthroscopy	No significant difference	Clinical and arthroscopic outcomes of ASC implantation were encouraging for OA knees in both groups, although there were no significant differences in outcome scores between groups.
Bui et al. (2014) [26]	Adipose SVF (ASC) + PRP via percutaneous injections	Case series	21 OA	>18; ND	Various treatments without any success	None	8.5	VAS; functions; MRI	VAS/function/MRI improvements	ASC + PRP: effective in treating OA of knees
Michalek et al. (2015) [27]	Adipose SVF (ASC) via percutaneous injections	Multi-center case control study	1114 OA	Median 62 (range, 19–94); 589 M and 525 F	Various treatments without any success	None	Median 17.2	VAS; functions	VAS/function improvements	Adipose SVF is a novel and promising treatment approach for patients with degenerative OA. ASC is safe and cost-effective.
Fodor et al. (2016) [28]	Adipose SVF (ASC) percutaneous injections	Case report	6 OA	Mean 59 (range, 51–69); 1 M and 5 F	Various conservative treatments without any success	None	12	VAS; functions (WOMAC, ROM, TUG); MRI	VAS/function improvements but no MRI evidence of cartilage regeneration	Autologous adipose SVF is a safe and potential new therapy for pain reduction in knee OA.
Pak et al. (2013) [32]	Adipose SVF (ASC) + PRP via percutaneous injections	Case series	3 chondromalacia patellae	54; 1 M. 43 and 63; 2 F. All with chronic knee pain.	Various treatments without any success	None	3	VAS; functions (FRI, ROM); MRI	VAS/function improvements and MRI evidence of cartilage regeneration	ASC + PRP: effective in treating chondromalacia patellae patients
Pak et al. (2014) [37]	Adipose SVF (ASC) + PRP via percutaneous injections	Case report	1 patient with meniscus tear	32;1 F with chronic knee pain due to meniscus tear	Various treatments without any success	None	3	VAS; functions (FRI, ROM); MRI	VAS/function improvements and MRI evidence of cartilage regeneration	ASC + PRP: effective in treating cartilage defect lesions, including meniscus tear
Pak (2011) [11]	Adipose SVF (ASC) + PRP + HA via percutaneous injections	Case report	2 osteonecrosis of femoral head	47; 1 M. 29; 1 F.	Various conservative treatments without any success	None	3	VAS; functions (FRI, ROM); MRI	VAS/function improvements and MRI evidence of boneregeneration	ASC + PRP + HA: potentially effective in regenerating bone in humans
Pak (2012) [40]	Adipose SVF (ASC) + PRP + HA via percutaneous injections	Case report	2 osteonecrosis of femoral head	34 and 39; 2 M	Various conservative treatments without any success	None	7 and 16	VAS; functions (FRI, ROM); MRI	VAS/function improvements and MRI evidence of bone regeneration	Regenerated bone by using ASC + PRP + HA may persist, representing potential future therapy for osteonecrosis.
Pak et al. (2014) [41]	Adipose SVF (ASC) + PRP + HA via percutaneous injections	Case report	1 osteonecrosis of femoral head	43; 1 M	Various conservative treatments without any success	None	3	VAS; functions (FRI, ROM); MRI	VAS/function improvements and MRI evidence of bone regeneration	ASC + PRP + HA: potentially effective in regenerating bone in humans
Saxer et al. (2016) [42]	Adipose SVF (ASC) + ceramic granules + fibrin gel	Case report	8 proximal humeral fractures	68; IM. Mean 70.4 (range, 62–84); 7 F.	None	Open reduction and internal fixation	12	VAS; biopsies; mCT	VAS improvements; biopsies and mCT evidence of bone (tissue) regeneration	Spontaneous bone tissue and vessel formation within a fracture-microenvironment with autologous adipose SVF
de Girolamo et al. (2016) [49]	Adipose SVF (ASC) vs PRP	Randomized prospective clinical trial	56 patients with Achilles tendinopathy PRP group: 28; SVF group: 28	PRP group: 46.6 6.2; ND. SVF group: 47.3 3.8; ND	None	None	6	VAS; functions (VISA-A, AOFAS, SF-36); US and MRI	VAS, VISA-A, AOFAS, SF-36 improved and structural changes on US and MRI	Both PRP and adipose SVF are safe and effective for Achilles tendinopathy but adipose SVF yields faster results.
Lee et al. (2015) [50]	Allogeneic adipose ASC + fibrin glue	Open-label pilot study	12 patients with lateral epicondylosis	Mean 51.8 ± 9.5; 5 M and 7 F	None	None	13	VAS; functions (MEPI); US	VAS, MEPI improved; tendon defects improved	Allogeneic ASC is safe and effective in treating lateral epicondylosis

*SVF* stromal vascular fraction, *ASC* adipose tissue-derived stem cells, *PRP* platelet-rich plasma, *HA* hyaluronic acid, *ECM* extracellular matrix, *OA* osteoarthritis, *yr* year, *mo* month, *M* male, *F* female, *ND* not described, *MRI* magnetic resonance imaging, *mCT* microcomputed tomography, *US* ultrasonography, *HTO* high tibial osteotomy, *VAS* visual analogue scale, *FRI* functional rate index, *ROM* range of motion, *WOMAC* western Ontario and McMaster Universities osteoarthritis index, *Lysholm* Lysholm scores, *Tegner* Tegner activity scale, *TUG* Timed up-and-go, *MEPI* mayo elbow performance index, *AOFAS* American Orthopaedic Foot & Ankle Society, *SF-36* Short Form-36, *VISA-A* Victorian Institute of Sport Assessment for Achilles tendinopathy

## Autologous adipose SVF

### Preparation of autologous adipose SVF

In order to obtain autologous adipose SVF, liposuction is first performed. The resulting adipose tissue is called lipoaspirate. The lipoaspirates are then digested with collagenase to break down the matrix. Consequently, MSCs are released from the matrix of the adipose tissue [8, 9]. These MSCs are termed adipose tissue-derived stem cells (ASCs). Afterward, by using the centrifugation-and-dilution method, the ASCs are isolated and collagenase is washed off. After 3 to 4 rounds of centrifugation and dilution, the bottom few milliliters of the end-product are obtained. The end-product is considered to be SVF [8, 9]. Autologous adipose SVF contains a variety of cells: MSCs, pericytes, vascular adventitial cells, fibroblasts, pre-adipocytes, monocytes, macrophages, red blood cells, fibrous tissue, and extracellular matrix (ECM) [8, 9].

### Stem cells in autologous adipose SVF

The number of stem cells contained in the adipose SVF can fluctuate widely. In adipose tissue, the numbers of nucleated cells can range from 500,000 to 2,000,000 cells per gram (g) of adipose tissue, and 1 to 10% of these nucleated cells are considered to be ASCs [12]. The number of ASCs in 1 g of adipose tissue may vary from 5000 to 200,000 stem cells [12]. Theoretically, in 100 g of adipose tissue, 0.5–20 million ASCs can be extracted in the SVF form. One of the reasons for such variation can be attributed to individual differences. Different patients have different adipose tissue texture and density [13]. Some of the adipose tissue is denser than the other, probably due to different amount of ECM.

In addition to differences in individual adipose tissue, collagenase may also play an important role, affecting the yield and viability of stem cells in SVF. High dosage or prolonged exposure to collagenase may be toxic to stem cells. Thus, an excess amount of collagenase can decrease stem cell viability. However, insufficient amount of collagenase may result in an inefficient and inadequate amount of stem cell yield [14]. Thus, using the correct amount of collagenase is very important. In addition, the correct type of collagenase is just as important. There are numerous types of collagenase available commercially. Collagenase is produced by two separate and distinct genes in the bacterium *Clostridium histolyticum*. The *col*G gene codes for type I collagenase and the *col*H gene codes for type II collagenase. Various enzymes such as elastase, trypsin, and/or papain can be added to these two types of collagenase to increase the specificity for certain tissues [15].

## Current clinical applications of autologous adipose SVF in cartilage regeneration

### Cartilage regeneration in OA

OA is a debilitating health problem common in elderly patient populations worldwide. Painful OA lowers quality of life by limiting the normal daily activities of patients [16, 17]. Current existing medical treatments aim to remedy symptoms only. Commonly prescribed treatments include non-steroidal anti-inflammatory drugs (NSAIDs), steroids, hyaluronic acids (HAs), and physical therapy. However, MSCs, in the form of autologous adipose SVF or culture expanded form, are an alternative therapy that can potentially treat the underlying cause of OA by regenerating cartilage.

One of the major drawbacks of applying autologous adipose SVF in orthopedic conditions is the lack of availability of randomized controlled studies. Most, if not all, literature available with regard to the human application of autologous adipose SVF are either in the format of case reports or cohort studies. Due to such constraints, despite the successful results reported by these articles, it is not yet readily accepted as a mainstream medical treatment.

In 2011, for the first time, Pak reported a case series of treating patients with OA of the knees with autologous adipose SVF and regenerating cartilage-like tissue [11]. Pak obtained autologous adipose SVF from digesting about 100 g of adipose tissue with collagenase and going through the centrifugation-dilution washing cycle as described by Zuk et al. [8, 9]. This autologous adipose SVF, with platelet rich plasma (PRP) and HA, was then injected percutaneously into the knee joints of two patients. After 3 months, the visual analog score (VAS) for pain, functional rating index (FRI), and range of motion (ROM) of the patients were assessed and shown to be improved along with MRI evidence of cartilage regeneration [11]. The inclusion criteria and exclusion criteria were listed as follows: Inclusion criteria: (i) chronic or degenerative joint disease causing significant functional disability and/or pain; (ii) the failure of conservative treatments; and (iii) an unwillingness to proceed with surgical intervention. Exclusion criteria: (i) active inflammatory or connective tissue disease thought to impact pain condition (i.e., lupus, rheumatoid arthritis, and fibromyalgia); (ii) active non-corrected endocrine disorder that might impact pain condition (i.e., hypothyroidism and diabetes); (iii) active neurologic disorder that might impact pain condition (i.e., peripheral neuropathy and multiple sclerosis); (iv) pulmonary and cardiac disease uncontrolled with medication usage; (v) history of active neoplasm within the past 5 years; (vi) blood disorders documented by abnormal complete blood count (CBC) within 3 months including severe anemia, thrombocytopenia, leukocytosis and/or leukopenenia; and (vii) medical conditions precluding the injection procedures.

Subsequently in 2013, Pak et al. reported a retrospective cohort study involving 91 patients with various orthopedic conditions [18]. Between the period of 2009 and 2010, Pak et al. treated 91 patients with OA of the knees, OA of the hips, and osteonecrosis of the femoral heads with percutaneous injections of autologous adipose SVFs along with autologous PRPs and HAs. The study reported the average efficacy of the regenerative treatment to be 65% at 3 months without any serious side effects and without any development of tumors. Some of the side effects reported were swelling and tendonitis [18].

In 2016, Pak et al. published a case series reporting that addition of autologous adipose ECM along with the SVF may also be effective when used together with autologous PRP and HA [19]. As in other reports, Pak et al. obtained autologous adipose SVF from digesting 100 g of adipose tissue with a collagenase. However, this time, unlike other reports, they added autologous adipose tissue-derived ECM, extracted by using an adipose tissue homogenizer, into the mixture of autologous adipose SVF, along with autologous PRP and HA. The mixture was injected into the knees of three patients with OA of the knees. Three months after treatment, all three patients’ symptoms, measured using FRI, ROM, and VAS pain score, improved. In addition, comparison of pre-treatment and post-treatment MRI data of all three patients demonstrated cartilage-like tissue regeneration [19].

In 2012, Koh and Choi also reported a retrospective cohort study treating 25 OA patients with autologous adipose SVF with autologous PRP [20]. This group obtained autologous adipose SVF from digesting only 19 g of adipose tissue extracted from the knee fat pad. Koh et al. also used the centrifugation-dilution method described by Zuk et al. [8, 9]. As performed by Pak et al., these adipose SVFs with autologous PRP was percutaneously injected into the knees of 25 patients with OA after performing arthroscopic debridement and lavage. The article states that the mean Lysholm knee scoring scales, Tegner activity level scales, and VAS scores improved significantly in the treated group compared to the control group. No imaging studies were carried out. No major side effects were reported [20].

In 2013, Koh et al. reported a case series involving 18 patients with OA of the knees receiving autologous adipose SVF obtained from digesting only 9 g of adipose tissue from the knee fat pad [21]. The autologous adipose SVF with autologous PRP were percutaneously injected into knees of 18 patients after arthroscopic debridement and lavage. After a few months, the patients were evaluated with Western Ontario and McMaster Universities osteoarthritis index (WOMAC) scores, Lysholm knee scoring scales, and VAS scores and MRI studies. The patients improved on all criteria, including the cartilage whole-organ MRI scores. No serious complications were reported [21].

In 2014, Koh et al. reported a case series involving second-look arthroscopy results in 35 patients with knee OA treated with autologous adipose SVF [22]. In this report, Koh et al. incorporated arthroscopic guidance when injecting the knees with adipose SVF. Initially, the patients underwent arthroscopic examinations with debridement and lavage. Afterward, autologous adipose SVF with autologous PRP were injected under arthroscopic guidance. Only about 23 g of adipose tissue was used. About 12.7 months after treatment, second-look arthroscopy was performed. The results showed that the mean International Knee Documentation Committee (IKDC) and Tegner activity level scales significantly improved, but 76% of the patients had abnormal repair tissue observed during arthroscopy [22].

In another study reported by Koh et al. in 2014, the clinical results and second-look arthroscopy findings were compared between an autologous adipose SVF/PRP injection group and a PRP-only group [23]. This study involved 44 patients undergoing open-wedge high tibial osteotomies (HTO). This time, autologous adipose SVF were obtained from 120 g of adipose tissue from the patients’ buttocks. Afterward, the autologous adipose SVFs were injected with autologous PRP in 23 patients under arthroscopic guidance and the other 21 patients were injected with autologous PRP alone under arthroscopic guidance. After 24 months of the treatment, the results showed that the autologous adipose SVF/PRP group showed significantly greater improvement than the PRP-only group, as measured by VAS for pain, Knee injury Osteoarthritis Outcome Score (KOOS) subscales for pain and symptoms, and second-look arthroscopic evaluation. Arthroscopic exams showed fibrocartilage regeneration in 50% of the adipose SVF/PRP group versus 10% in the PRP-only group. However, the Lysholm score was similarly improved in both groups [23].

Later in 2015, Koh et al. reported another case series involving second-look arthroscopy results of 30 patients with OA of the knees treated with autologous adipose SVF obtained from 120 g of adipose tissue from the patients’ buttocks [24]. The autologous adipose SVF was injected with PRP under arthroscopic guidance. Of the 30 patients, 16 patients underwent second look arthroscopies about 25 months after the initial treatment. Of the 16 patients, 10 patients (63%) had improved cartilage, 4 patients (25%) had maintained the cartilage, but 2 patients (12%) failed in healing cartilage defects. The study reported that all patients showed significant improvement in OA outcome scores (KOOS), VAS pain scale, and Lysholm score [24].

In another study, Kim et al. compared the efficacy of autologous adipose SVF alone to that of autologous adipose SVF with fibrin glue [25]. The fibrin glue was used as a scaffold for stem cells to attach. This study involved 54 patients with knee OA. Autologous adipose SVF was obtained from digesting 120 g of adipose tissue with collagenase. Of the 54 patients, 37 patients were treated with autologous adipose SVF only and the other 17 patients were injected with autologous adipose SVF with fibrin glue. After about 28 months, the mean IKDC score and Tegner activity level scale in both groups were compared and had improved significantly; the improvement was comparable in both groups. However, in second-look arthroscopies, International Cartilage Repair Society (ICRS) scores were better in the adipose SV with fibrin glue group [25].

In 2014, Bui et al. reported a case series involving 21 patients with OA of the knees [26]. The patients were treated with autologous adipose SVF with PRP. The adipose SVF was obtained from digesting 50–100 ml of lipoaspirates originating from the abdomen. Then, the autologous adipose SVF with autologous PRP was injected percutaneously into the diseased knees. After 8.5 months of treatment, all 21 patients showed improved VAS pain score and the Lysholm score. There was also a significant increase in the thickness of the cartilage, as depicted on the MRIs [26].

In early 2015, Michalek et al. reported a multi-center case-control study involving 1114 patients with OA of the knees and hips from four different countries (USA, Czech Republic, Slovakia, and Lithuania) [27]. These patients were percutaneously injected with autologous adipose SVF and followed for average 17 months. No serious side effects were reported and no incidents of cancer were reported. The clinical effects, measured on the basis of pain, non-steroid analgesic usage, limping, extent of joint movement, and stiffness, all improved. At 12 months after treatment, 63% of all patients reported approximately 75% symptom improvement and 91% of all patients reported approximately 50% of symptom improvement [27].

In 2016, Fodor et al., another group in the USA, reported clinical improvement of 8 knee OA patients treated with autologous adipose SVF obtained by digesting 150–250 ml of lipoaspirates [28]. All patients attained full activity with decreased knee pain. WOMAC scores, VAS pain scale score, ROM, and timed up-and-go (TUG) results all improved. The improvement in WOMAC scores and VAS scores were maintained at 1 year. Comparing preoperative MRI to 3-months postoperative MRI showed no detectable structural differences. No major side effects were observed [28].

### Chondromalacia patellae (CMP)

CMP is a knee joint disorder defined by cartilaginous softening of patellar bone cartilage and may cause patellofemoral pain syndrome (PFPS), which is characterized by anterior knee pain (AKP) along with malalignment of the tibio-patello-femoral joint [29, 30]. CMP can be diagnosed with MRI along with clinical history and physical examination [29, 30]. Currently, only symptomatic treatment is available. As in OA, commonly prescribed treatments include NSAIDs and physical therapy. Thus, CMP poses a major therapeutic challenge. However, as a few recent studies have shown the possibility of cartilage recovery using MSCs [31], the combination of autologous adipose SVF with correction of alignment may be a novel approach to treating CMP.

In 2013, Pak et al. reported a case series involving three patients with CMP of the knees [32]. Pak et al. treated these patients with autologous adipose SVF using 100 g of adipose tissue obtained from the abdomen of the patients. The adipose SVF was injected percutaneously with PRP and HA. After 3 months of treatment, the patients’ symptoms improved in terms of VAS pain scale, FRI, and ROM. The study also showed positive regeneration of hyaline cartilage at the patellofemoral joints of all three patients between pre- and post-treatment MRIs [32].

### Meniscus tear

The meniscus is a fibrocartilaginous disk that functions to transfer weight, absolve shock to the knee, and to protect the hyaline cartilage at the knee joint [33]. With knee injuries, the meniscus may be damaged causing it to be torn. Such meniscus tears are initially treated conservatively with NSAIDs and physical therapy [34, 35]. If conservative treatment fails, an arthroscopic meniscectomy is traditionally performed. However, arthroscopic meniscectomy, either full or partial, is associated with early onset of OA of the knees [36]. Thus, potential cartilage regeneration with MSCs, or autologous adipose SVF, may offer a major therapeutic breakthrough.

In 2014, Pak et al. reported that autologous adipose SVF may be effective in treating meniscus tears [37]. This case report involved one patient treated with autologous adipose SVF obtained from digesting approximately 40 g of packed adipose tissue with collagenase. Afterward, the autologous adipose SVF was injected with PRP and HA. After 3 months of treatment, the patient’s symptoms, measured with VAS scores for pain, FRI, and physical therapy ROM, had improved. In addition, probable regeneration of the meniscus cartilage was documented by pre- and post-treatment MRIs [37].

## Current clinical applications of autologous adipose SVF in bone regeneration

Bone has an innate capability to regenerate. Upon fracture, resident progenitor stem cells work to form scarless healing [38]. However, a few clinical instances require therapeutic interventions to facilitate bone repair and regeneration.

### Osteonecrosis of the femoral head

Osteonecrosis of the femoral head is a debilitating skeletal disorder of unknown etiology that usually occurs in young males, can lead to collapse of the hip joint and may necessitate a total hip replacement [39].

In 2011, Pak reported that autologous adipose SVF may have the capability to regenerate bone in the lesion of osteonecrosis of the femoral head [11]. Pak obtained autologous adipose SVF from digesting 100 g of adipose tissue with collagenase. This autologous adipose SVF was then injected percutaneously with PRP and HA into hip joints of two patients. After 3 months, VAS for pain, FRI, and ROM of the hips were improved, and there was MRI evidence of bone regeneration [11].

Subsequently in 2012, Pak reported the long-term effect of autologous adipose SVF on bone regeneration in patients with osteonecrosis of the femoral head [40]. Of the two patients involved, one patient was followed for 7 months and the other patient for 16 months. The patients’ symptom improved and the MRI showed positive bone regeneration in both patients. Both patients clearly showed maintenance of the regenerated bone for a relatively long time period [40].

In another case report, Pak et al. treated a patient with stage 1 osteonecrosis of femoral head with autologous adipose SVF [41]. Pak et al. obtained adipose SVF from digesting 100 g of adipose tissue with collagenase. The autologous adipose SVF with PRP and HA was injected into the femoral head under ultrasound guidance. Three months after the injection, patient’s symptom completely resolved and the MRI findings of necrosis resolved completely as well. A subsequent MRI taken a few months later showed maintenance of the regenerated bone [41].

### Bone fracture

In a case report by Saxer et al. in 2016, autologous adipose SVF was used with ceramic granules within fibrin gel to treat proximal humeral fractures in conjunction with standard open reduction and internal fixation in eight patients [42]. Up to 12 months after the procedure, biopsies of the repair tissue were performed and demonstrated formation of bone ossicles that were structurally disconnected and morphologically distinct from osteoconducted bone, which suggests the osteogenic nature of implanted SVF cells. This study demonstrated spontaneous bone tissue and vessel formation within a fracture microenvironment with autologous adipose SVF [42].

### Non-union fracture

Although autologous adipose SVF may be indicated for treatment of a non-union fracture, there have not been any reports so far.

## Current clinical applications of autologous (or allogeneic) adipose SVF in tendon/ligament regeneration

For patients with chronic tendinopathy, conservative medical management, including anti-inflammatory drugs, physiotherapy, braces, and therapeutic exercises, has produced unsatisfactory outcomes [43, 44]. Although corticosteroid injection has been widely used for short-term pain relief, the effectiveness of the treatment is transient [45, 46]. In addition, by suppressing the cellular activity of human tenocytes and collagen synthesis, corticosteroid injections weaken the tendon, thereby increasing the risk of rupture [46, 47]. Injection approaches with dextrose solutions, whole blood, and platelet-rich plasma have been tried with limited evidences of success [48]. Potential regenerative MSC therapy, on the other hand, is emerging as a novel treatment for chronic tendinopathy.

### Achilles tendinopathy

In 2016, de Girolamo et al. reported a result of randomized prospective clinical trial involving 56 patients with Achilles tendinopathy [49]. Of the 56 patients, 28 patients were randomly assigned to a single autologous PRP injection and the other 28 patients were assigned to a single autologous adipose SVF injection. All patients were assessed clinically using VAS, Victorian Institute of Sport Assessment for Achilles tendinopathy (VISA-A), the American Orthopaedic Foot & Ankle Society (AOFAS) and Short Form-36 (SF-36) forms. Before the treatments, all patients also underwent ultrasound imaging studies and MRIs; these were then repeated at 4 and 6 month follow-ups. At the final follow-up, both patients group showed significant improvements in all scores compared to baseline (*p* < 0.05). In the adipose SVF injection patients, these improvements were faster and more pronounced. After 6 months, the MRI and ultrasound studies showed no significant difference. No side effects were observed in either group. The study concluded that both PRP and SVF are safe and effective treatments for Achilles tendinopathy, although adipose SVF may allow faster clinical results than PRP [49].

### Lateral epicondylosis

Lee et al. published an article in 2015 involving 12 patients with lateral epicondylosis treated with allogeneic adipose-derived MSCs [50]. Although the scope of this article is limited to autologous adipose SVF, the study by Lee et al. is significant in light of the fact that an insufficient number of human studies are available with regards to tendon and ligament repair. The study is a pilot study assessing the safety and efficacy of culture expanded ASCs in treating human patients with lateral epicondylosis. The ASCs were injected with fibrin glue under ultrasound guidance into the hypoechoic tendon lesions of chronic lateral epicondylosis. Then, patients’ VAS score, modified Mayo clinic performance index, and longitudinal and transverse ultrasound images of the tendon defect areas were evaluated at 6, 12, 26, and 52 weeks. Through 52 weeks of follow-up, VAS scores progressively decreased and elbow performance scores improved. Tendon defects, assessed by ultrasound images, also significantly decreased throughout the follow-up period. No significant adverse effects were observed [50].

## Discussion

Due to the current regulatory environment, culture expanded MSCs are considered to be a pharmaceutical product and require governmental clearance and approval. Autologous adipose SVF injection, on the other hand, is considered to be a medical procedure, and thus allowed in many parts of the world. Consequently, autologous adipose SVF is slowly being tried as an alternative treatment in the field of orthopedics, treating disorders involving cartilage, bone, and tendons/ligaments. Compared to bone marrow SVF, adipose tissue is considered to be a preferred source of MSCs in the form of SVF due to its ease of accessibility and the availability of a large number of stem cells per gram of adipose tissue [12].

Although numerous studies available that show the effectiveness of autologous adipose SVF treatment in OA patients, the comparison of these studies show lack of standardization. Lacking standardization may lead to differences in results of the treatment. Most of the standardization may be improved with availability of culture expanded stem cells [50, 51]. With differences of procedure in processing adipose tissue, the yield of viable stem cells may differ from one group to the other. However, with the availability of culture expanded stem cells, all variables that exist in the manual process may be eliminated, providing consistent quantity and quality of stem cells. With the standardized availability of culture expanded stem cells, the effectiveness would become more improved.

In addition, it should be well known that OA, CMP, and meniscus tear are all diseases of the joint, not just cartilage. In these joint problems, cartilage, ligaments, tendons, muscles, and bone are all involved. For example, CMP involves alignment of the knee. In patients with CMP, correction of only cartilage may not dramatically improve the symptoms unless the misalignment is also corrected. As for meniscus tear is involved, improving muscles, tendon, and ligament may also be important in addition to cartilage regeneration.

It seems the amount of autologous adipose tissue used in producing adipose SVF has no direct relationship with the efficacy and safety observed. Some of the studies used only 20 g of adipose tissue while others have used more than 100 g of adipose tissue. However, Jo el al. showed in a double-blind randomized clinical trial that higher number of stem cells may result in improved cartilage regeneration [51].

Although number of ASCs contained in autologous adipose SVF should play an important role in regenerative medicine, other components in the adipose SVF may also play important roles. Autologous adipose SVF contains various cells including ASCs and ECM [11, 12]. It is well known that ECM excretes a variety of cytokines and growth factors [52–54]. In addition, ECM may work as a scaffold, assisting ASCs to adhere to the lesion [55].

As Zuk et al. showed in 2001 and 2002, ASCs in the adipose SVF have the capacity to regenerate bone, cartilage, muscle, and adipose tissue. Likewise, human data is accumulating in the field of orthopedics that ASCs contained in adipose SVF can be applied to treat various disorders by regenerating cartilage and bone. Recently, a study clearly showed that regeneration of a tendon in a human patient is possible with autologous adipose SVF. As shown by de Girolamo et al. [49], and to a certain extent by Lee et al. [50] since this group used culture expanded ASCs, adipose SVF can be used to treat tendon injuries. These results may be used to further extrapolate that adipose SVF and MSCs may be used in the treatment of ligament injuries.

Although the successful applications of autologous adipose SVF in humans may represent a promising, minimally invasive, non-surgical alternative, many issues (challenges and limitations) need to be resolved and clarified before the general application of this procedure in clinics. Firstly, how ASCs in the form of SVF may help joint diseases remains unclear: (i) it could be due to the secretory effects of the stem cells injected [56, 57]; (ii) it could be due to direct engraftment and differentiation of the stem cells that were introduced into the diseased joints [58, 59]; or (iii) it could be due to the combination of secretory effects and direct engraftment of the stem cells. Adipose stem cells excrete a variety of cytokines, chemokines, growth factors, and exosomes [60, 61]. These factors have positive effects on the surrounding progenitor cells. However, there are some evidence that these stem cells injected may actually become engrafted into the tissue and differentiate into tissue-specific stem cells [62]. It is also very possible that these two mechanisms play a role in cartilage regeneration.

Secondly, how long can ASCs or SVF (after injection) stay in the joint before they are cleared out? To the best of our knowledge, most of fluid is reabsorbed within few days after the injection of SVF. However, the fate of ASCs injected into a joint is not yet clear. It can be assumed that ASCs may stay in the joint and be attached to the lesion via scaffold. ASCs that are attached and integrated may be able to survive prolonged period of time. However, it can be assumed that ASCs that are not integrated into the tissue may die slowly while excreting various trophic factors.

Thirdly, is proper control (e.g., control group only receiving saline washes but not stem cells) needed in order to make a fair conclusion that the effect of SVF seen here is due to SVF injection but not the washout of inflammatory cytokines by saline? Since it is possible that PRP may have a regenerative potential, three are studies comparing PRP to autologous SVF to confirm the regenerative effects of SVF [20, 23, 49]. However, it is necessary to have a controlled study comparing saline control group to autologous SVF group to confirm the real effect of SVF.

Fourthly, whether would the quality of ASCs affect the therapeutic effect? For example, will ASCs from obese vs non-obese patients have similar results of healing? It is very well known that people have different texture of adipose tissue as well as differences in adipose cell size [63]. Thus, the lipoaspirate tissue must be different in different individuals. Since the lipoaspirate processing step, including the dosage of the collagenase, is usually constant within the treating group, the end result of the different tissue must yield difference adipose SVF. Therefore, it is very probable that there may be some differences in quantity and quality of ASCs in obese and non-obese patients. Compared with ASCs from non-obese individuals, ASCs from obese individuals have showed increased proliferation and migration capacity but decreased differentiation capacity [64]. Multiple studies have documented the reduction in the osteogenic differentiation capacity of ASCs in obese individuals [65–67]. Therefore, there is a need for investigating whether ASCs from obese vs non-obese patients have similar results of healing of human orthopedic disorders.

Lastly, are all the cell types contained in SVF beneficial for disease healing? The adipose SVF contains numerous cells types, including red blood cells (RBCs), white blood cells (WBCs), adipocytes, along with MSCs [8, 9]. In addition, the adipose SVF may contain left-over collagenase, which can cause connective tissue damage, as it is being used to breakdown the connective tissue in the adipose tissue. These extra cells (RBCs and WBCs), either intact or fragmented, may elicit other responses. It is probable that the joint swelling after injecting the autologous adipose SVF may be due to these extra cells and/or collagenase contained in the SVF [18].

## Conclusions

Autologous adipose SVF, containing MSCs that are termed ASCs, has a great clinical potential to treat various orthopedic disorders as seen in human studies. Along with autologous adipose SVF, double-blind, randomized human clinical trials are being conducted using culture expanded MSCs with promising results. Until culture expanded stem cells are available for various orthopedic applications, autologous adipose SVF may be worthwhile to try in individuals for whom medical treatment has failed and for whom surgical options are not available.

## Abbreviations

AKP: Anterior knee pain
AOFAS: American Orthopaedic Foot & Ankle Society
ASC: Adipose tissue-derived stem cell
CMP: Chondromalacia patellae
ECM: Extracellular matrix
FRI: Functional rating index
HA: Hyaluronic acid
HTO: High tibial osteotomies
ICRS: International Cartilage Repair Society
IKDC: International Knee Documentation Committee
KOOS: Knee injury Osteoarthritis Outcome Score
MEPI: Mayo elbow performance index
MRI: Magnetic resonance imaging
MSC: Mesenchymal stem cell
NSAID: Non-steroidal anti-inflammatory drug
OA: Osteoarthritis
PFPS: Patellofemoral pain syndrome
PRP: Platelet rich plasma
ROM: Range of motion
SF-36: Short Form-36
SVF: Stromal vascular fraction
TUG: Timed up-and-go
VAS: Visual analog score
VISA-A: Victorian Institute of Sport Assessment for Achilles tendinopathy
WOMAC: Western Ontario and McMaster Universities osteoarthritis index
